# Analytical Model for the Duty Cycle in Solar-Based EH-WSN for Environmental Monitoring

**DOI:** 10.3390/s18082499

**Published:** 2018-08-01

**Authors:** Sebastià Galmés, Soledad Escolar

**Affiliations:** 1 Department of Mathematics and Computer Science, University of Balearic Islands, 07122 Palma de Mallorca, Spain; sebastia.galmes@uib.es; 2 School of Computing Engineering, University of Castilla-La Mancha, 13071 Ciudad Real, Spain

**Keywords:** wireless sensor network, energy consumption model, duty cycle, low power listening, MAC, TinyOS, energy harvesting model, solar irradiance

## Abstract

A technology drift is currently taking place from traditional battery-powered sensor networks, which exhibit limited lifetime, to the new Energy-Harvesting Wireless Sensor Networks (EH-WSN), which open the way towards self-sustained operation. However, this emergent modality also brings up new challenges, especially due to the time-varying nature and unpredictability of ambient energy sources. Most proposals for implementing EH-WSN rely on heuristic approaches to redesign the duty-cycling mechanism at the MAC layer, with the ultimate goal of optimizing network performance while preserving self-sustained and continuous operation. In contrast to the common system-wide reduced duty cycle of battery-powered sensor networks, the duty cycle in EH-WSN is much larger and adapted to the energy harvesting rate and traffic load of each node in the network. In this paper, we focus on solar-based EH-WSN devoted to environmental monitoring. In contrast to current works, we follow an analytical approach, which results into closed-form expressions for the duty cycle and initial energy storage that guarantee self-sustained operation to any node in a solar-based EH-WSN. To center the analysis, we consider TinyOS sensor nodes, though we postulate that the essential components of the obtained formulation will contribute to further develop duty cycle adaptation schemes for TinyOS and other software platforms.

## 1. Introduction

A wireless sensor network is a wireless network of low-cost, low-power and small-in-size multifunctional nodes, which have limited sensing, processing and communicating capabilities and cooperate with each other to relay sensed data from a region of interest to one or multiple sinks. From its emergence in the early nineties, the main design goal faced by researchers in this area has been network lifetime. This is due to the fact that, for many years, it has been assumed that sensor nodes are battery-powered, and so it has become essential to develop solutions aimed at optimizing energy consumption. These solutions span all components of a sensor node and, in the case of the communication component, all layers of the protocol stack. However, the main research efforts have been devoted to the design of energy-aware MAC (Medium Access Control) protocols, since major sources of energy consumption take place at this layer; channel-sensing, packet transmission and reception, packet overhearing, collisions and idle listening. However, in contrast to channel-sensing, packet transmission and reception, packet overhearing and collisions, which represent energy expenses on the scale of the relatively short packet duration, idle listening is especially harmful, because a node might spend a great amount of time listening to the channel without receiving any packet. This is particularly true in the case of event-driven sensor networks designed for rare event detection, and also in the case of time-driven sensor networks supporting environmental monitoring applications, where reporting times are typically much larger than the duration of packets. Hence, in order to reduce the impact of idle listening, the majority of MAC protocols for battery-powered sensor networks are based on duty-cycling the communication activity (these protocols are described in more detail in the next section). Nevertheless, even the best of such protocols cannot avoid node batteries to become exhausted after a finite (and relatively short) period of time. On the other hand, the new vision of Internet of Things (IoT), from which WSN (Wireless Sensor Networks) will become an integral part, claims enhanced energy management mechanisms that lead to much longer network lifetimes while providing QoS (Quality Of Service) guarantees. To achieve this goal, the scientific and industrial communities have recently intensified their efforts towards the introduction of energy-harvesting technologies in wireless sensor networks, with the ultimate goal of taking self-sustained WSN from vision to reality. The size compatibility between harvesting devices and the small sensor nodes has been and continues being one of the main obstacles, since the efficiency of the former is directly proportional to their surface or volume. Fortunately, research efforts have made it possible nowadays to develop various cost-effective energy scavenging mechanisms tailored to the intrinsic characteristics of sensor nodes. Among them, photovoltaic cells constitute the most widely deployed alternative in spite of the unavailability of solar power during night times or under bad weather conditions. This is due to their higher energy conversion efficiency compared to other mechanisms, such as vibrational, electromagnetic, thermoelectric, wind, etc. References [1,2,3,4,5] provide good surveys on energy scavenging mechanisms for EH-WSN.

However, size compatibility between harvesting devices and sensor nodes is not the only challenge faced by researchers in EH-WSN. In fact, this new paradigm poses additional and even more difficult challenges due to the time-varying nature and unpredictability of most energy sources. As stated in [1], the main research focus is again on the MAC layer, but according to a fundamental difference in the design principle of EH-WSN compared to their battery-powered counterpart: whereas the latter were developed with the objective of maximizing network lifetime, the energy-harvesting paradigm allows for paying more attention to other design objectives, namely performance metrics such as throughput and delay, in line with the high expectations from IoT. Note that QoS was already taken into account in the last generation of MAC protocols for battery-powered sensor networks (see, for instance, [6,7]), but subject to rigid energy budget constraints. Contrarily, in EH-WSN the emphasis is put on network performance while ensuring a sustainable energy state (more formally, this is known as energy neutral operation, which means that, in a period of time, the energy drained from the environment is equal to or larger than the energy consumed by the node). Thus, it is of crucial importance to consider the energy-harvesting mechanism in the design of MAC protocols for EH-WSN; otherwise, it is not possible to provide performance guarantees under self-sustained operation [1]. Currently, such redesign process has already produced various MAC protocols for EH-WSN. These protocols again rely on duty-cycling the communication activity, but allowing for much larger duty cycles which, in turn, can be individually adapted to the energy harvesting and consumption rates of each node. The work presented in [1] proposes a taxonomy of duty-cycled MAC protocols for EH-WSN based on the element that triggers the communication process (sender, receiver or sink). This taxonomy and the most relevant protocols in each category are also examined in the next section.

As it will be noticed, a common feature of current MAC protocols for EH-WSN is that they are based on heuristic approaches. Since the main design objective is network performance under self-sustained operation, these approaches focus on dynamically adjusting the duty cycle of nodes according to their energy level. So, nodes with more available energy increase their duty cycle in order to enhance throughput and/or delay, whereas those experiencing energy scarcity switch to sleep mode (to recharge batteries), thus diminishing their traffic activity and causing some performance degradation. However, the mathematical relationship between node duty cycle and other parameters such as energy harvesting rate and traffic load remains unknown. This is more or less successfully overcome by current protocols by imposing a tight control of the energy available at nodes via frequent measurements. Alternatively, in this paper, we follow an analytical approach, which allows for obtaining closed-form expressions for the duty cycle and initial energy storage that guarantee self-sustained operation at each node. This does not only contributes to a better understanding the dependence of node duty cycle on energy-related and traffic-related parameters, but we expect that it will result in helping the development of simpler and more effective adaptive schemes for adjusting the duty cycle of nodes. To fix ideas, we consider a widely deployed duty-cycled MAC protocol, which is the LPL (Low Power Listening) mechanism implemented in TinyOS sensor nodes, though the essential results can be extrapolated to other software (and corresponding hardware) platforms. We assume a typical environmental monitoring application, where nodes periodically sense and transmit towards a base station via multi-hop communication (time-driven paradigm). Based on these assumptions, we first derive an accurate model for the energy consumed by sensor nodes during each period of the communication process (communication round). Then, by using an experimentally tested theoretical model for the energy harvested by solar cells, we formulate the condition for energy neutral operation, from which we obtain the above mentioned closed-form expressions in terms of known and/or measurable parameters.

More specifically, the contributions of this paper can be listed as follows:

- We develop an analytical model for the energy consumed (per round) by sensor nodes supporting the LPL mechanism implemented in TinyOS. With no loss of generality, we consider the MicaZ [8] hardware platform integrating the CC2420 radio in the evaluation, as it has been one of the most widespread commercial solutions. In addition, this selection allows for validating the resulting model via simulation with Avrora [9]. Avrora is a very accurate and highly scalable sensor node simulator that offers a complete framework for evaluating applications written in TinyOS 1.x/2.x for the Mica family nodes, which include an AVR microcontroller.
- We develop an analytical model for the duty cycle of nodes in terms of relevant parameters describing their traffic load and energy harvesting capability. This model results from the formulation of the condition for energy neutral operation and the assumption that solar irradiance follows a periodical pattern.
- We show that, in general, duty cycle adjustment is not enough to support non-disrupted operation, but an initial level of energy is also necessary. So, in addition, the proposed model includes a closed-form expression for the energy that is initially required for self-sustained operation.

To the best of our knowledge, the work presented in this paper represents the first attempt to tackle the characterization of the node duty cycle for energy neutral operation in an analytical way. The rest of the paper is organized as follows. In Section 2, we revise the state of the art on MAC protocols for battery-powered and energy-harvesting WSN. Then, in Section 3, we describe the LPL mechanism implemented in TinyOS for duty cycling the communication activity of sensor nodes. In Section 4, we construct an analytical model for the energy consumed by any node in a TinyOS WSN supporting an active-monitoring (time-driven) application. In Section 5, we validate the proposed model via simulation. Section 6 describes an accurate model for energy harvesting based on solar cells. Then, by using both the energy consumption and energy harvesting models, in Section 7 we develop closed-form expressions for the duty cycle and initial energy storage that guarantee energy neutral operation. Finally, in Section 8, we draw the main conclusions and suggestions for further research.

## 2. Related Work

Current MAC protocols for battery-powered sensor networks can be classified according to the type of duty cycle implementation. Accordingly, the following categories are devised:

- Contention-free, schedule-based or synchronous MAC protocols. The idea of these protocols is to couple the duty cycles of sender and receiver, in such a way that both wake up at the same time in order to exchange one or several packets. To achieve this behavior, a predetermined or negotiated schedule is used, which requires tight synchronization among nodes. This represents a disadvantage from the implementation point of view, but even more severe is the fact that such a rigid schedule leads to limitations in terms of network scalability, protocol adaptability to spatial and temporal variations, robustness, etc. Altogether, these deficiencies make scheduled protocols less attractive in spite of their benefits: very low duty cycles and absence of collisions and packet overhearing. These protocols rely on the principles of TDMA (Time Division Multiple Access). Some examples are Self-Organizing MAC (SMACS) [10], Sensor-MAC (SMAC) [11], ReSync [12], TRaffic-Adaptive Medium Access protocol (TRAMA) [13] and Timeout-MAC (TMAC) [14].
- Contention-based, random access or asynchronous MAC protocols. In this case, clock synchronization is avoided and consequently the duty cycles of nodes are completely decoupled. Thus, in order to link a transmitter that has data to send to a receiver that is duty-cycling, many asynchronous protocols make use of LPL techniques. Essentially, as stated in [15], these techniques shift the burden of synchronization to the sender. In the most primitive form of LPL, a transmitter sends a sufficiently long preamble before the data packet, in such a way that, upon waking up, the receiver detects the preamble and stays awake for the time required to capture the packet. This implementation, known as preamble sampling (PS), represents a solution purely focused on the physical layer. Examples of protocols using preamble sampling are Aloha with preamble sampling [16] and B-MAC [17]. Later, more efficient implementations were developed, which basically substituted the long preamble by a repetitive sequence of a wake-up packet, which could be the data packet itself or an advertisement packet. The resulting protocols could then be categorized according to the way the receive check is performed: either at the MAC layer (packet header recognition), as in X-MAC [15], or at the physical layer (energy detection), as in BoX-MAC-1 and BoX-MAC-2 [18]. Finally, an alternative to LPL is a technique called Low Power Probing (LPP) [19]. In this case, it is the receiving node that periodically sends small packets called beacons or probes, to announce that it is awake and ready to receive data. A node willing to send a packet turns its radio on and waits for a probe. Upon receiving a probe from the intended destination, it sends an acknowledgement (ACK) and, subsequently, the data packet. The most representative LPP-based protocols are Receiver-Initiated MAC (RI-MAC) [20] and A-MAC [21]. An extensive survey on asynchronous MAC protocols for wireless sensor networks is provided in [22].
- Hybrid protocols. In general, asynchronous protocols offer better balance among multiple design criteria than their synchronous counterparts. Certainly, despite asynchronous protocols are less energy-efficient due to their contention nature, they exhibit higher levels of scalability, flexibility and robustness. In order to keep the benefits of the two categories, some hybrid solutions have also been proposed over the years, like WiseMAC [23] or Zebra MAC (Z-MAC) [24]. As stated in [18], these approaches attempt to combine TDMA with certain types of asynchronous support. However, these protocols have not led to standard implementations.
- Adaptive protocols. Other protocols focus on dynamically setting the duty cycle of nodes so as to make it adaptive to changing traffic conditions. This can be done over synchronous or asynchronous/hybrid solutions, though the latter have attracted most proposals. The duty cycle can be adjusted either by modifying the duty period or the sleep period (or both), under a variety of criteria that span several QoS metrics as well as energy efficiency. For instance, some protocols dynamically adjust the duty cycle based on the traffic load and/or topology information in order to achieve acceptable balances between QoS parameters (throughput, latency, reliability) and energy consumption. This is the case of BoostMAC [25], MaxMAC [26], Scheduled Channel Polling MAC (SCP-MAC) [27], Energy-Aware Adaptive LPL (EA-ALPL) [28], ASLEEP [29], Demand Wakeup MAC (DW-MAC) [30] and, more recently, DISSense [31] and Cross Layer Adaptation of Check Intervals (CLAC) [32]. Some of these protocols are in fact mechanisms built on top of other known protocols. For instance, EA-ALPL is built on top of B-MAC, and CLAC is implemented on top of BoX-MAC-2 and the Collection Tree Protocol (CTP) [33]. These two protocols are, respectively, the MAC and network layer solutions adopted in TinyOS/MicaZ nodes. Other contributions proposing adaptive duty cycles are [34,35,36].

More information on MAC protocols for battery-powered wireless sensor networks can be found in [6,7,37,38,39,40,41]. However, it is commonly accepted that these protocols are inappropriate under the energy-harvesting paradigm. As stated in the previous section, the primary objective in the design of EH-WSN is to optimize performance while preserving self-sustained operation. EH-WSN are subject to the time-varying nature of energy sources, fact that may result into periods of insufficient energy along the network lifecycle. For this reason, MAC protocols for EH-WSN try to couple traffic activity in transmission-reception pairs with peaks of energy in the participating nodes, while at the same time minimizing the impact on delay or throughput. Whereas these new requirements make MAC protocol design even more complex, the benefits in terms of longer network lifetimes or perpetual operation are invaluable. As stated in [1], synchronous MAC protocols are not deemed appropriate for EH-WSN because tight schedules are not compatible with the temporal and spatial variability of energy sources. Thus, the focus is on asynchronous protocols. The authors in [1] classify asynchronous MAC protocols for EH-WSN into three categories depending on the element that initiates the communication process: sink-initiated, receiver-initiated and sender-initiated asynchronous protocols. Next, these categories are described in more detail.

In sink-initiated asynchronous MAC protocols, it is the sink that triggers the communication process by polling sensor nodes. Particularly, in [42] a probabilistic polling approach is adopted, which takes into account the unpredictability of energy sources. More specifically, the sink sets a contention probability in every polling packet to indicate the probability that any sensor node in receiving state should send its data packet. Upon receiving the polling packet, a sensor node generates an internal random number and decides to transmit the data packet if the generated number is below the contention probability; otherwise, the node remains in receiving state or switches to charging state if its residual energy falls below a predetermined value. Ideally, only one out of the set of sensor nodes in receiving state should transmit after having received the polling packet; precisely, the core algorithm executed by the sink consists of adapting the contention probability from poll to poll in order to achieve this goal. This approach is formulated in [42] for single-hop WSN; then, its multi-hop extension is proposed in [43]. In [44], an adaptive MAC protocol that maintains energy efficiency and quality of service for an IEEE 802.15.4 standard-based IoT network is proposed. The core contribution is an algorithm that dynamically adjusts the sleeping period of nodes in order to allow them for harvesting sufficient RF energy from a surrounding LTE eNodeB. Finally, another sink-initiated MAC protocol is AH-MAC (Adaptive Hierarchical MAC) [45], which is suitable for low-rate large-scale wireless sensor networks supporting active monitoring applications or event-driven alarm systems. AH-MAC combines the benefits of LEACH [46] and IEEE 802.15.4 to implement a scalable, self-configurable and self-healing wireless sensor network that incorporates energy-harvesting at a reasonable cost: only predetermined cluster heads are equipped with energy-harvesting circuits in AH-MAC. Accordingly, the activity of regular battery-powered nodes is limited to uploading their data to the cluster heads, which support most network tasks.

In receiver-initiated asynchronous MAC protocols, it is every receiver that requests data transmission from its senders. For instance, in the EH-MAC protocol proposed in [47], a receiver uses a probabilistic polling mechanism to request data packets. Compared to deterministic polling, this mechanism is more consistent with the unpredictable state of potential senders, given the temporal and spatial variability of the energy that can be harvested from the environment. Moreover, the contention probability value contained in the polling packet is dynamically adjusted by the receiver in order to reduce data packet collisions and maximize network throughput. Note that EH-MAC is very similar to the protocol proposed in [43], with the main difference that the polling activity in EH-MAC is transferred to the receivers. Another receiver-initiated MAC protocol is ODMAC (On-Demand MAC) [48], which exploits the fact that typically sensor networks are low traffic networks. In ODMAC the communication is on demand, meaning that a sensor node transmits a packet only upon being requested by a receiver. To do this, receivers periodically broadcast a beacon frame to indicate senders that they are ready to receive. Senders only wake up when they have queued packets to be transmitted to the sink; after waking up, they listen to the channel waiting for a beacon and may enter a contention period if other senders react to the same beacon. So, the burden of communication (idle listening) is transferred from receivers to transmitters, fact that benefits the overall energy balance because most of time transmission queues are empty (low traffic condition). Additionally, each receiver dynamically adjusts the beacon period according to the energy profiles of its sending nodes. As a result, the per-node duty cycle is kept as large as possible to maximize performance while, at the same time, energy neutral operation is preserved. LEB-MAC (Load and Energy Balancing MAC) [49] follows a similar approach, but in this case a receiving node stamps the next time it will wake up with a certain probability in the beacon message. This probability is locally computed based on recent energy-harvesting history, and thus it takes into account the variability of environmental factors. Upon receiving a beacon message, sending nodes can synchronize their duty cycles with the receiver duty cycle as long as they have sufficient energy. So, the resulting duty cycles and node throughputs are consistent with current energy states. ODMAC and LEB-MAC do not avoid contention between multiple senders trying to transmit data to the same receiver. In contrast, the authors of QAEE-MAC (QoS-Aware Energy Efficient) [50] propose an exchange of beacon messages between senders and receivers in order to reduce contention. Specifically, if a sender node wants to transmit a data packet, it first sends a Tx-beacon and waits for a beacon from the intended receiver (Rx-beacon). For its part, the receiver periodically wakes up and listens to the channel to receive all sender beacon frames. Next, this node determines which sender can transmit first (decision process that may include priorities to distinguish between normal and urgent data), and broadcasts a receiver beacon containing the identity of the selected sender as well as a network allocation vector (NAV) indicating the next wakeup time of the receiver. Moreover, the NAV value can be adjusted by the receiver according to its own energy profile. After capturing a beacon message, all senders except the one selected by the receiver switch to sleep mode, thus guaranteeing contention avoidance. It is shown that this protocol improves throughput, especially in the case of critical data.

The last subset of MAC layer solutions for EH-WSN falls into the category of transmitter-initiated asynchronous protocols. One example is DeepSleep [51], which consists of a MAC enhancement scheme on the baseline IEEE 802.11 power saving mode (PS). DeepSleep was developed in the light of the forthcoming M2M (Machine To Machine) networks, which are expected to consist of large amounts of energy-harvesting devices. Large number of devices means high level of contention in the original IEEE 802.11, and hence high energy expenditure in idle listening, packet overhearing and collisions. Whereas this can be supported by personal devices such as mobile telephones and portable computers, as they can be recharged frequently, it is not deemed appropriate for autonomous wireless networks featuring long operation periods. Basically, DeepSleep introduces two enhancements: it grants higher channel access priority to devices with lower energy levels (this is done by reducing their contention window during backoff intervals), and it randomly defers the wakeup times of high-priority devices in order to reduce contention among them. In summary, DeepSleep improves performance (outage probability, packet loss rate and transmission delay) under self-sustained operation. Another solution is the EL-MAC (Energy Level MAC) protocol proposed in [52], which assumes that energy-harvesting devices are secondary users in a cognitive radio network. In essence, EL-MAC benefits low-energy nodes by allowing all nodes to compute an access probability and a contention window size on the basis of their residual energy. The access probability defines the probability that a node wishing to transmit decides to sense the channel; if not, channel-sensing is postponed and the node switches to sleep mode. The contention window size is the maximum number of CSMA/CA contention slots selected by the node once it has decided to sense the channel and transmit. Obviously, secondary users with lower energy levels will compute higher access probabilities and smaller contention windows. Finally, another relevant contribution in this category is the RF-MAC protocol [53], which also retains the essential concepts of CSMA/CA. RF-MAC adapts CSMA/CA to the specificities of sensor nodes that harvest radio frequency (RF) energy. The design is based on a experimental study that demonstrates how the location and number of RF transmitters, as well as the chosen frequency, impact node charging time. As in previous protocols, some parameters of the CSMA/CA mechanism are dynamically varied in order to balance energy delivery to sensor nodes with overall communication performance. Examples of these parameters are the slot time, the inter-frame spacing and the contention window size.

As stated in the previous section, the protocols just described are based on heuristic approaches to the problem of optimizing network performance under self-sustained operation. In contrast, in this paper we follow an analytical approach. In the next section, we start by developing an accurate energy consumption model for the LPL mechanism implemented in TinyOS sensor nodes.

## 3. Background

The default MAC protocol delivered with the TinyOS 2.x release [54,55] for the MicaZ platform, which holds a CC2420 radio [56], consists of an implementation of a protocol known as BoX-MAC [18]. This implementation, called BoX-MAC-2, was jointly proposed with implementation BoX-MAC-1 by David Moss and Philip Levis in [18]. Thus, this section aims to describe the operation of BoX-MAC-2, as it becomes a major issue in order to understand the energy model proposed in the next section.

**Figure 1:**
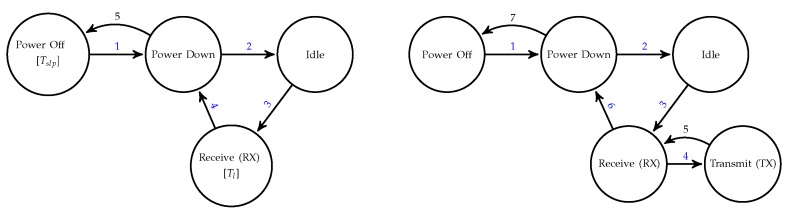
State transition diagrams of one CC2420 radio activation with no channel activity (**left**) and with channel activity (**right**). RX and Power Off states include also their durations, $T_{l}$ and $T_{slp}$, respectively, on the figure on the left.

The CC2420 radio exhibits five operational states with different energy consumptions: (1) Power Off, where the voltage regulator is switched off; (2) Power Down, where the voltage regulator is on but the on-chip oscillator is still turned off; (3) Idle, where the oscillator is running; (4) Receive (RX), for reception mode; and (5) Transmit (TX), for transmission mode. Transitions between these states are triggered as consequence of some action on the radio. In essence, the LPL TinyOS mechanism alternately switches the CC2420 radio between the states ON and OFF, though through several intermediate states. For example, when a sensor node wakes up and puts its radio on to listen the channel, three transitions take place: (1) from Power Off to Power Down, (2) from Power Down to Idle, and (3) from Idle to RX. Analogously, when the sensor node turns off the radio from RX state, transitions from RX to Power Down and from Power Down to Power Off occur. Specifically, the state transition diagram for a CC2420 radio activation in which no ongoing packets are heard by the node is shown in Figure 1 on the left: the radio transits from Power Off to RX (passing through the intermediate states) and remains in the latter state for a period of time $T_{l}$; after that, the radio transits from RX to Power Off and stays in this state for $T_{slp}$ in order to perform a complete sleep period. Alternately, it could be the case that the node listens to an in-progress packet over the channel. This could occur during any radio activation of the node, at some instant between the start and end of a predetermined listening period. If this occurs, the listener keeps its radio at RX state until the packet is completely received and, after that, it transits to TX state in order to send the corresponding acknowledgement to the sender. Note that, in general, the time for checking the channel will be smaller than the duration of the RX period ($T_{l}$). Note also that the transmitter could need several retries until the receiver catches the full packet. Additionally, as the receiver of the packet will be generally a forwarding node (except in the case of the base station), this will perform subsequent TX-RX transitions until receiving an acknowledgement from its next hop. Then, before switching to the Power Off state, the node listens to the channel again for an additional time called DELAY_AFTER_RECEIVE, whose purpose is to keep the radio in RX state when activity, either transmission or reception, is detected on the channel. Note that, although the term suggests that this time is only spent after a reception occurs, it is also spent after a transmission occurs (DELAY_AFTER_RECEIVE in CC2420 Low Power Listening: http://mail.millennium.berkeley.edu/pipermail/tinyos-help/2008-May/033858.html). Figure 1 on the right shows the state transition diagram for this scenario. Finally, it could also happen that the reception (and subsequent forwarding) of a packet and the transmission of a packet generated by the node itself coincide at the same radio activation. In this case, the node behaves as explained previously and, additionally, it must accomplish the transmission of the own packet, for which the node goes into a new loop of TX-RX transitions until receiving a new ACK from its next hop.

In summary, $T_{l}$ can be interpreted as the nominal duration of the RX state when there is no channel activity, or, in other words, as the minimum amount of time that the radio is kept on (active) after each activation (the so-called DUTY_ON_TIME in TinyOS context). Note that the maximum time of activity depends on the traffic load and thus it is a priori unknown (the LPL TinyOS mechanism maintains the radio on beyond the value $T_{l}$ when it detects channel activity). Regarding $T_{slp}$, this is the nominal duration of the sleep period (Power Off state), that is, the time elapsed between a radio deactivation and the next radio activation. On the other hand, for the setup of the LPL mechanism, the developer has to fix a duty cycle (DC) to be used by all sensor nodes. This is a well-known MAC-layer parameter that represents the level of activity of a sensor node in communication. It is established statically according to the frequency with which the application performs environmental sensing and packet transmission. Based on DC, the MAC layer configures the sleep period, whose duration can be calculated as $T_{slp}=\frac{T_{l}\left(100-DC\right)}{DC}$. Note that, according to this expression, DC is the percentage that the minimum radio activation period represents with regard to the total period; hence, it refers to activation periods with no channel activity. Both parameters, $T_{l}$ and $T_{slp}$, and their relationship through DC, represent a simplified view of the communication module as alternating between only two states, active (ON) and inactive (OFF), with no transitory states. This is precisely the perspective adopted in the construction of the energy consumption model in the next section. This is not only a totally acceptable approximation, but it also helps to make the analytical model less dependent on the particular radio module.

## 4. Analytical Energy Consumption Model

In this section we derive an analytical model for the energy consumed by a sensor node that is part of a data-gathering tree for environmental monitoring. We assume that this node supports the LPL implementation embedded in TinyOS. Its task consists of receiving and forwarding packets from other nodes as well as generating and transmitting own packets containing local readings. Given the lack of synchronization between any transmitting node and its corresponding receiver, the time lag between the start of their duty periods is completely random. Hence, energy consumption will also be subject to randomness, what means that we can expect a model of stochastic nature. In fact, the analysis that follows involves the derivation of complete statistical distributions for the two basic sources of energy consumption in a sensor network, namely transmission and reception. Next, the problem is addressed in detail.

### 4.1. Assumptions

In order to preserve the analytical tractability, the energy model developed in this section relies on some simplifying assumptions, which will be further validated via the simulation tests described in the next section. These assumptions are as follows:

- The sensor network supports a monitoring application, which means that nodes periodically sense the environment and send the corresponding data towards a sink or base station. So, the network behaves as a data-gathering tree where the overall data flow from nodes to base station is organized into communication rounds whose duration is known as reporting period. With no loss of generality, we assume that every node generates a single packet per communication round (which may result from aggregating multiple samples of the environment).
- Power control is enabled and hence every node can tune its transmission power according to the receiver distance (in fact, this is why we define forward and backward transmission distances).
- For simplicity purposes, the proposed model does not reflect the transitory states described in Figure 1 (Power Down and Idle), which means that it only captures the alternation between RX-TX (ON) and Power Off (OFF) states. However, given the small durations and current draws that correspond to transitory states, this assumption can be adopted without penalizing the realistic nature of the model. In fact, simulation results shown in Section 5 reveal the validity of this assumption.
- There are no packet collisions. Collisions are quite unpredictable, as they depend on the spatial and temporal distribution of the traffic load; however, given the fact that nodes are not synchronized and typically reporting periods in data-gathering applications are very large (on the order of minutes or more), we can reasonably assume that, after an initial period, transmissions become sufficiently randomized so as to neglect collisions. Again, the experiments performed with Avrora (reported in the next section) allowed us to definitely validate this assumption.
- Since transmissions are randomized, we can also assume that all packets processed by any intermediate node during a communication round (either generated or received and forwarded from any other node) occupy different duty cycles. This assumption is based again on the fact that reporting periods in monitoring applications are very large compared to the length of duty cycles. The experiments performed with Avrora showed that, even if this is not the case for some nodes, it only affects the way energy consumption is distributed over time, but it has very little impact on the overall balance of energy consumption per node and per communication round.

### 4.2. Analytical Model

**Figure 2:**
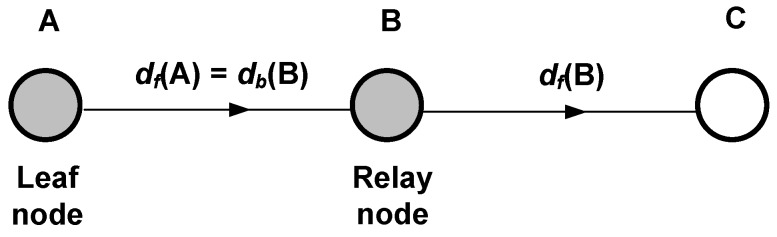
Scenario under analysis: node *A* generates and transmits a packet and node *B* receives and forwards this packet to its next-hop node.

**Figure 3:**
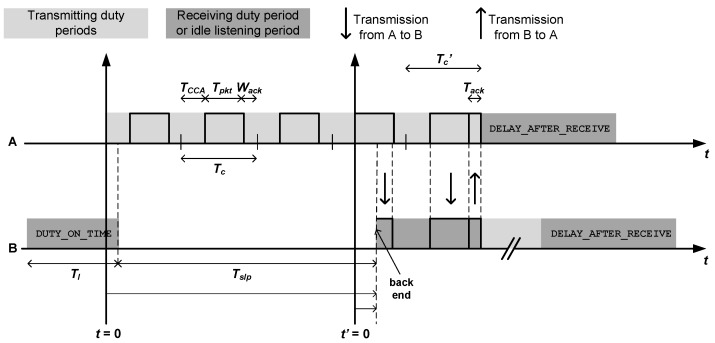
Time diagram for packet generation and transmission (node A) and packet forwarding (node B). The transmission process starts at the beginning of a duty period in the sending node A. This period is extended to the total time required to complete the transmission, i.e., until an ACK is received from node B. Packet reception by node B generally involves detection of a packet fragment before the complete packet is received and acknowledged. Then, the node forwads the packet through its next-hop link. Note also that the two nodes spend an extra time in reception mode before switching again to sleep mode.

Under the above assumptions, our modelling process relies on two fundamental calculations: the energy wasted by a node to transmit a single packet, and the energy wasted by a node to receive and forward a single packet too. For this purpose, we start by considering the scenario shown in Figure 2, where node A is a leaf node that generates and transmits a packet and node B is a relay or intermediate node that receives and forwards the packet from A. As it can also be noticed, we distinguish between forward and backward transmission distances in order to account for the general case where power control is enabled. In general, for an arbitrary node *X*, $d_{f}\left(X\right)$ is the distance between node *X* and the node to which it transmits packets (forward transmission distance), whereas $d_{b}\left(X\right)$ is the distance between node *X* and a node from which it receives packets (backward transmission distance). For the detailed analysis, we can use the time diagram shown in Figure 3, which also helps to understand the operation of the LPL TinyOS mechanism in a transmitter-receiver pair. Based on this diagram, the next two subsections are devoted to the analysis of the energy wasted by nodes A and B in their respective roles. Then, we develop a general expression for the energy wasted by an arbitrary node that receives and forwards multiple packets from other nodes, in addition to generating and transmitting an own packet. Since the results take form of statistical distributions, in the last subsection we obtain the corresponding expectations.

#### 4.2.1. Packet Generation and Transmission

As it can be noticed from Figure 3, there are some relevant time intervals in addition to the already defined $T_{l}$ (DUTY_ON_TIME), $T_{slp}$ and DELAY_AFTER_RECEIVE:

- $T_{c}$: This is the transmission cycle, which is the time spent by a node in the transmission of a single non-successful packet. During this period, three standard operations are performed in sequence: (1) clear channel assessment (CCA) and random back-offing ($T_{CCA}$); (2) packet transmission ($T_{pkt}$); and (3) waiting for acknowledgement ($W_{ack}$). In general, the first time component is an unpredictable variable that depends on the amount of workload and its spatio-temporal distribution over the network. Thus, it is usually characterized by its average. However, if transmissions from all nodes are sufficiently randomized, as we have postulated in our assumptions, the channel is likely to be found idle every time a node senses the channel. Hence, the incurred time is just for clear channel assessment and thus deterministic and of small value ($T_{CCA}$ constant and small).
- $T_{c}^{\prime }$: This is a modified transmission cycle that corresponds to the transmission of a successful packet. It is very similar to the previous one, but instead of including a waiting time for acknowledgement, it contains the actual time to receive such acknowledgement.
- $T_{ack}$: This is the duration of acknowledgements packets.

In order to ensure that a receiving node, upon its radio activation, detects the start of a packet transmission regardless of the asynchrony with the sender, it must be accomplished that $T_{l}>T_{c}$.

From Figure 3 we can notice that in general the transmitter needs to send its packet repeatedly until the receiver catches it entirely after waking up from its sleep period. Depending on the number of tries, the transmitter wastes more or less energy. Thus, we first characterize the number of tries (*k*) required by the transmitter to receive an acknowledgement from the receiver. Table 1 shows the characterization of this random variable by taking the backend of the receiver duty period as reference (see Figure 3). Note that the actual position of such backend is measured with regard to the time origin set up in the figure, which is the start of the transmitter duty period. Accordingly, for every outcome of the number of tries, the table shows the time interval to which the backend belongs, as well as its probability. As noticed, the case of single try corresponds to the union of two time intervals: in the first one, the level of asynchrony between transmitter and receiver is very small, whereas in the second one such asynchrony is sufficiently large so as to make the next receiver duty period come into scene. Also, it is worth observing that the overall distribution is not uniform because not all time intervals are equally long; in particular, the probability of a single try is precisely the duty cycle of the LPL mechanism. Finally, α is an auxiliary parameter defined as follows:

(1) $$\begin{array}{c} \alpha =\left[\frac{T_{slp}}{T_{c}}\right] \end{array}$$

Then, if node A requires *k* tries to successfully send a packet to B, the energy wasted to exclusively complete the transmission of such packet obeys the following equation:

(2) $$\begin{array}{c} E_{T}\left(A\right)=\left(k-1\right)E_{C}\left(A\right)+E_{C}^{\prime }\left(A\right)+E_{l}^{DAR} \end{array}$$

In this expression, $E_{C}\left(A\right)$ is the energy wasted by node A in any non-successful transmission cycle, $E_{C}^{\prime }\left(A\right)$ is the energy wasted in the last (successful) transmission cycle, in which node A actually receives an acknowledgement, and $E_{l}^{DAR}$ is the energy wasted in idle listening during the final DELAY_AFTER_RECEIVE period. More specifically, we have:

(3) $$\begin{array}{c} E_{C}\left(A\right)=E_{l}^{CCA}+E_{tx}^{pkt}\left(d_{f}\left(A\right)\right)+E_{l}^{ack} \end{array}$$

(4) $$\begin{array}{c} E_{C}^{\prime }\left(A\right)=E_{l}^{CCA}+E_{tx}^{pkt}\left(d_{f}\left(A\right)\right)+E_{rx}^{ack} \end{array}$$

Here $E_{l}^{CCA}$, $E_{tx}^{pkt}\left(d_{f}\left(A\right)\right)$, $E_{l}^{ack}$ and $E_{rx}^{ack}$ are respectively the energy losses experienced by node A to sense the channel, transmit a single packet to B, wait for the corresponding acknowledgement and actually receive such acknowledgement.

#### 4.2.2. Packet Forwarding

Figure 3 also describes the temporal breakdown of the process of receiving and forwarding a packet (by node B). In general, upon waking up, node B detects a fragment of the transmitted packet before its complete reception. Of course, this fragment may not exist if the wake up takes place during the gap between two consecutive tries. As stated in Section 3, once the packet is completely received, the node issues an acknowledgement, forwards the packet, and enters a DELAY_AFTER_RECEIVE period before switching to sleep mode. Let us start by calculating the energy wasted by node B to complete the reception of the packet from A (that is, until it transmits the acknowledgement packet):

(5) $$\begin{array}{c} E_{R}\left(B\right)=E_{fd}+E_{rx}^{pkt}+E_{tx}^{ack}\left(d_{b}\left(B\right)\right) \end{array}$$

Here, $E_{fd}$ denotes the energy cost of the fraction of duty period that precedes the full packet reception; in other words, it is the energy wasted during the time elapsed between the wakeup of the receiver and the start of the successful packet reception. Additionally, $E_{rx}^{pkt}$ and $E_{tx}^{ack}\left(d_{b}\left(B\right)\right)$ are, respectively, the energy costs experienced by node B to receive and confirm the packet from A. Among all such energy components, the only one that is subject to randomness is $E_{fd}$, as it depends on the asynchrony between the two nodes. For the characterization of this component, we set up another time origin at the beginning of the last non-successful packet transmitted by node A. Accordingly, $t^{\prime }$ represents the shift of the backend of the duty period of node B with regard to the new reference; this shift may vary from $0^{+}$ to $T_{c}^{-}$ (see again Figure 3). More specifically, *t* and $t^{\prime }$ are related through the following equation:

(6) $$\begin{array}{c} t=T_{CCA}+\left(k-2\right)T_{c}+t^{\prime },k=2\ldots \alpha +1 \end{array}$$

**Figure 4:**
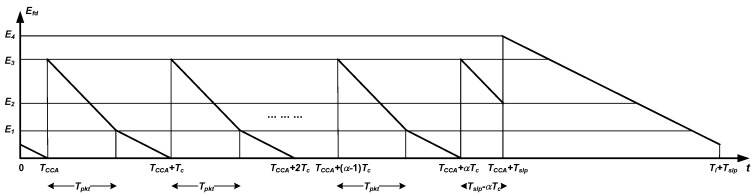
Evolution of $E_{fd}$ in terms of the transmitter-receiver asynchrony, for $T_{slp}\leq \alpha T_{c}+T_{pkt}$.

**Figure 5:**
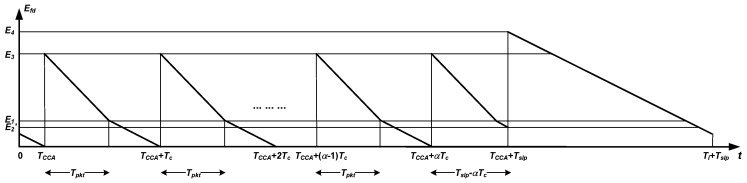
Evolution of $E_{fd}$ in terms of the transmitter-receiver asynchrony, for $T_{slp}\geq \alpha T_{c}+T_{pkt}$.

Based on this equation, Table 2 provides a characterization of $E_{fd}$ in terms of the transmitter-receiver asynchrony. A new magnitude appears, namely $E_{l}$, which is the total energy wasted in idle listening during a radio activation with no ongoing packets, the duration of which is precisely DUTY_ON_TIME. For illustration purposes, Figure 4 and Figure 5 plot the information contained in Table 2. In particular, Figure 4 corresponds to $T_{slp}\leq \alpha T_{c}+T_{pkt}$, whereas Figure 5 describes the opposite case (note that these two cases are distinguished in Table 2). Note also that the two graphs exhibit a similar sawtooth-like profile, only differing in the interval $\left[T_{CCA}+\alpha T_{c},T_{CCA}+T_{slp}\right)$. The change of slope that takes place in most subdomains of the two representations reflects the general case where power consumption in idle listening differs from that in packet reception. Otherwise, a single straight line would have been drawn. For the evaluation of the distribution of $E_{fd}$, some reference values have been indicated in Figure 4 and Figure 5:

(7) $$E_{1}=E_{l}\frac{W_{ack}+T_{CCA}}{T_{l}}\;$$

(8) $$E_{2}=E_{rx}^{pkt}\frac{T_{pkt}-\left(T_{slp}-\alpha T_{c}\right)}{T_{pkt}}+E_{l}\frac{W_{ack}+T_{CCA}}{T_{l}}\;$$

(9) $$E_{2}^{\prime }=E_{l}\frac{T_{c}-\left(T_{slp}-\alpha T_{c}\right)}{T_{l}}\;$$

(10) $$E_{3}=E_{rx}^{pkt}+E_{l}\frac{W_{ack}+T_{CCA}}{T_{l}}\;$$

(11) $$E_{4}=E_{l}$$

Note that it has been assumed that $E_{4}>E_{3}$ in Figure 4 and Figure 5. This is true if power consumption in idle listening is equal to the power consumption in packet reception. However, if this is not the case, it could happen that $E_{4}<E_{3}$ for sufficiently larger values of the power consumption in packet reception with regard to that in idle listening.

Next, based on the knowledge of $E_{fd}$ as a function of the transmitter-receiver asynchrony, and the fact that this asynchrony is uniformly distributed between 0 and $T_{l}+T_{slp}$, we can derive the cumulative distribution function of $E_{fd}$ viewed as a compound random variable. Let this distribution be F(y), that is, $F\left(y\right)=prob\left(E_{fd}\leq y\right)$. The result is as follows:

- Case 1: $T_{slp}\leq \alpha T_{c}+T_{pkt}$
  (12) $$\begin{array}{c} F\left(y\right)=\left\{\begin{array}{cc} \left(\alpha +1\right)\frac{y}{E_{l}}\frac{T_{l}}{T_{l}+T_{slp}} & \mathrm{if}\;0<y\leq E_{1}\\ F\left(E_{1}\right)+\frac{y-E_{1}}{T_{l}+T_{slp}}\left(\alpha \frac{T_{pkt}}{E_{rx}^{pkt}}+\frac{T_{l}}{E_{l}}\right) & \mathrm{if}\;E_{1}<y\leq E_{2}\\ F\left(E_{2}\right)+\frac{y-E_{2}}{T_{l}+T_{slp}}\left(\left(\alpha +1\right)\frac{T_{pkt}}{E_{rx}^{pkt}}+\frac{T_{l}}{E_{l}}\right) & \mathrm{if}\;E_{2}<y\leq E_{3}\\ F\left(E_{3}\right)+\frac{y-E_{3}}{E_{l}}\frac{T_{l}}{T_{l}+T_{slp}} & \mathrm{if}\;E_{3}<y\leq E_{4} \end{array}\right. \end{array}$$
- Case 2: $T_{slp}>\alpha T_{c}+T_{pkt}$
  (13) $$\begin{array}{c} F\left(y\right)=\left\{\begin{array}{cc} \left(\alpha +1\right)\frac{y}{E_{l}}\frac{T_{l}}{T_{l}+T_{slp}} & \mathrm{if}\;0<y\leq E_{2}^{\prime }\\ F\left(E_{2}^{\prime }\right)+\left(\alpha +2\right)\frac{y-E_{2}^{\prime }}{E_{l}}\frac{T_{l}}{T_{l}+T_{slp}} & \mathrm{if}\;E_{2}^{\prime }<y\leq E_{1}\\ F\left(E_{1}\right)+\frac{y-E_{1}}{T_{l}+T_{slp}}\left(\left(\alpha +1\right)\frac{T_{pkt}}{E_{rx}^{pkt}}+\frac{T_{l}}{E_{l}}\right) & \mathrm{if}\;E_{1}<y\leq E_{3}\\ F\left(E_{3}\right)+\frac{y-E_{3}}{E_{l}}\frac{T_{l}}{T_{l}+T_{slp}} & \mathrm{if}\;E_{3}<y\leq E_{4} \end{array}\right. \end{array}$$

Recalling again Figure 3, we can now set up the following equation for the total energy wasted by node B to forward a single packet from A ($E_{F}\left(B\right)$):

(14) $$\begin{array}{c} E_{F}\left(B\right)=E_{R}\left(B\right)+E_{T}\left(B\right) \end{array}$$

Here, $E_{T}\left(B\right)$ obeys Equation (2), but particularized for node B.

### 4.3. Energy Consumption per Round

**Figure 6:**
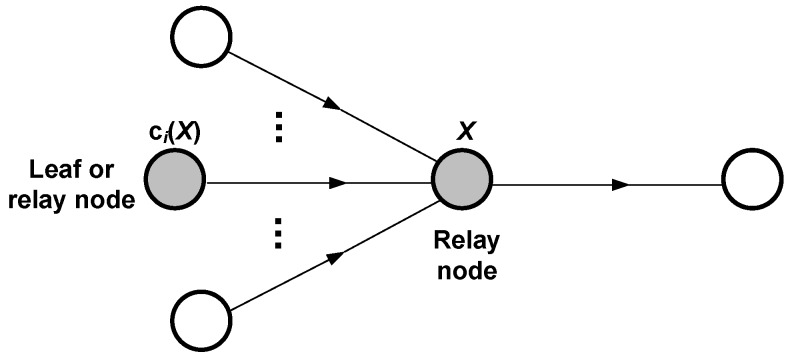
General scenario for the evaluation of the energy consumption per round.

The previous analysis has focused on the generation and transmission as well as the reception and forwarding of single packets. However, during a communication round, a node in a data-gathering tree is generally entailed to forward packets from multiple nodes in addition to transmitting its own packet. The scenario is described in Figure 6, where an arbitrary node *X* receives and forwards packets from all of its children. Every child is denoted by $c_{i}\left(X\right)$, with *i* varying between 1 and the total number of children, namely CH(X). Let also σ(X) denote the total number of descendants of node *X* (what includes its children and, recursively, the descendants of its children). Then, according to the assumption that transmissions become sufficiently randomized, in such a way that every packet managed by a node occupies a distinct duty period (recall Section 4.1), we can set up the following equation for $E_{round}\left(X\right)$, the total energy consumed by node *X* during a communication round:

(15) $$\begin{array}{ccc} E_{round}\left(X\right) & = & \sum_{i=1}^{CH(X)}\left(\sigma \left(c_{i}\left(X\right)\right)+1\right)E_{R,i}\left(X\right)+\left(\sigma \left(X\right)+1\right)E_{T}\left(X\right)\\ & + & \left(\left|\frac{T_{rnd}}{T_{l}+T_{slp}}\right|-\left(\sigma \left(X\right)+1\right)\right)\left(E_{l}+E_{sleep}\right) \end{array}$$

In this equation, $E_{R,i}\left(X\right)$ denotes the energy wasted by node *X* to receive a packet from its child node $c_{i}\left(X\right)$, whereas the last term accounts for the total energy wasted in duty cycles that are not dedicated to transmit or receive (unused duty cycles). Note that the energy wasted in sleep periods has been included: despite power consumption is very low during such periods, the overall energy consumption may be non-negligible if $T_{slp}\gg T_{l}$ (for low duty cycles). Also note that Equation (15) includes two types of random components: the number of tries *k* required by node *X* to upload its packets (contained in the component of energy wasted in transmission) and the fraction of duty period previous to successful reception from every child node (contained in every component of energy wasted in reception). Altogether, $E_{round}\left(X\right)$ contains CH(X)+1 random variables, which are mutually independent according to the randomization assumption. In the next subsection we compute the expected value of $E_{round}\left(X\right)$.

#### Expected Energy Consumption per Round

The stochastic nature of $E_{round}\left(X\right)$ has ultimately its origin in the random asynchrony between the duty periods of the two endpoints of any communication pair. In particular, the randomness of the number of tries (*k*) results from the randomness of the asynchrony between a node and its parent node (the node to which it transmits packets), whereas the randomness of $E_{fd}$ is due to the smaller-scale randomness (characterized by $t^{\prime }$ in Figure 3) between a node and any node from which it receives packets (child node). Accordingly, the evaluation of the expected energy consumption per round relies on averaging *k* and $E_{fd}$. In particular, from Table 1 we can easily derive the following expression for the average number of tries:

(16) $$\varepsilon \left[k\right]=\frac{\frac{\alpha }{2}\left(\alpha +3\right)T_{c}+\left(\alpha +2\right)\left(T_{slp}-\alpha T_{c}\right)+T_{l}}{T_{l}+T_{slp}}\;$$

For the calculation of the expectation of $E_{fd}$, we can use either its description in terms of the transmitter-receiver asynchrony provided in Table 2, jointly with the fact that this asynchrony is uniformly distributed, or its characterization in terms of the distribution function given in Equations (12) or (13). Having adopted the first alternative, we introduce the following auxiliary functions in order to simplify the analysis:

(17)Efd(1)(t′)=ErxpktTpkt−t′Tpkt+ElWack+TCCATl,t′∈[0,Tpkt)(18)I1(x)=∫0xEfd(1)(t′)dt′,x∈[0,Tpkt)(19)Efd(2)(t′)=ElTc−t′Tl,t′∈[Tpkt,Tc](20)I2(x)=∫TpktxEfd(2)(t′)dt′,x∈[Tpkt,Tc]

Accordingly, we have:

(21) $$\begin{array}{ccc} \varepsilon [E_{fd}] & = & \int_{0}^{T_{l}+T_{slp}}E_{fd}\left(t\right)\frac{1}{T_{l}+T_{slp}}\;dt\\ & = & \frac{1}{T_{l}+T_{slp}}\left[\int_{T_{CCA}}^{T_{CCA}+T_{c}}E_{fd}\left(t\right)\;dt+\ldots +\int_{T_{CCA}+\left(\alpha -1\right)T_{c}}^{T_{CCA}+\alpha T_{c}}E_{fd}\left(t\right)\;dt\right.\\ & & \left.+\int_{T_{CCA}+\alpha T_{c}}^{T_{CCA}+T_{slp}}E_{fd}\left(t\right)\;dt+\int_{0}^{T_{CCA}}E_{fd}\left(t\right)+\int_{T_{CCA}+T_{slp}}^{T_{l}+T_{slp}}E_{fd}\left(t\right)\;dt\right]\\ & = & \frac{1}{T_{l}+T_{slp}}\left[\alpha \left(I_{1}\left(T_{pkt}\right)+I_{2}\left(T_{c}\right)\right)+\int_{T_{CCA}+\alpha T_{c}}^{T_{CCA}+T_{slp}}E_{fd}\left(t\right)\;dt\right.\\ & & \left.+\int_{0}^{T_{CCA}}E_{fd}\left(t\right)+\int_{T_{CCA}+T_{slp}}^{T_{l}+T_{slp}}E_{fd}\left(t\right)\;dt\right] \end{array}$$

Note that the integral has been initially decomposed into the periodic component, which covers the region $\left[T_{CCA},T_{CCA}+T_{c}\right)\;\cup \ldots \cup \;\left[T_{CCA}+\left(\alpha -1\right)T_{c},T_{CCA}+\alpha T_{c}\right)$, and the rest of subdomains, namely $\left[0,T_{CCA}\right)$, $\left[T_{CCA}+\alpha T_{c},T_{CCA}+T_{slp}\right)$ and $\left[T_{CCA}+T_{slp},T_{l}+T_{slp}\right]$. Further development of $\varepsilon [E_{fd}]$ requires considering the two cases distinguished in Table 2 (for k=α+2):

- Case 1: $T_{slp}\leq \alpha T_{c}+T_{pkt}$
  (22) $$\begin{array}{c} \int_{T_{CCA}+\alpha T_{c}}^{T_{CCA}+T_{slp}}E_{fd}\left(t\right)\;dt=\int_{0}^{T_{slp}-\alpha T_{c}}E_{fd}^{\left(1\right)}\left(t^{\prime }\right)\;dt^{\prime }=I_{1}\left(T_{slp}-\alpha T_{c}\right) \end{array}$$
- Case 2: $T_{slp}>\alpha T_{c}+T_{pkt}$
  (23) $$\begin{array}{ccc} \int_{T_{CCA}+\alpha T_{c}}^{T_{CCA}+T_{slp}}E_{fd}\left(t\right)\;dt & = & \int_{0}^{T_{pkt}}E_{fd}^{\left(1\right)}\left(t^{\prime }\right)\;dt^{\prime }+\int_{T_{pkt}}^{T_{slp}-\alpha T_{c}}E_{fd}^{\left(2\right)}\left(t^{\prime }\right)\;dt^{\prime }\\ & = & I_{1}\left(T_{pkt}\right)+I_{2}\left(T_{slp}-\alpha T_{c}\right) \end{array}$$

On the other hand, the two last integrals in Equation (21) can be combined into a single integral from $T_{CCA}+T_{slp}$ to $T_{CCA}+T_{l}+T_{slp}$ and developed as follows:

(24) $$\begin{array}{c} \int_{T_{CCA}+T_{slp}}^{T_{CCA}+T_{l}+T_{slp}}E_{fd}\left(t\right)\;dt=\int_{T_{CCA}+T_{slp}}^{T_{CCA}+T_{l}+T_{slp}}E_{l}\frac{T_{l}+T_{slp}+T_{CCA}-t}{T_{l}}\;dt=\frac{E_{l}T_{l}}{2} \end{array}$$

Next, we can introduce expressions Equations (22) or (23) and (24) into the intermediate result given by Equation (21). In summary, we have:

- Case 1: $T_{slp}\leq \alpha T_{c}+T_{pkt}$
  (25) $$\begin{array}{c} \varepsilon \left[E_{fd}\right]=\frac{1}{T_{l}+T_{slp}}\left[\alpha \left(I_{1}\left(T_{pkt}\right)+I_{2}\left(T_{c}\right)\right)+I_{1}\left(T_{slp}-\alpha T_{c}\right)+\frac{E_{l}T_{l}}{2}\right] \end{array}$$
- Case 2: $T_{slp}>\alpha T_{c}+T_{pkt}$
  (26) $$\begin{array}{c} \varepsilon \left[E_{fd}\right]=\frac{1}{T_{l}+T_{slp}}\left[\left(\alpha +1\right)I_{1}\left(T_{pkt}\right)+\alpha I_{2}\left(T_{c}\right)+I_{2}\left(T_{slp}-\alpha T_{c}\right)+\frac{E_{l}T_{l}}{2}\right] \end{array}$$

Finally, the analysis can be completed by evaluating the auxiliary integrals:

(27) $$I_{1}\left(T_{pkt}\right)=E_{rx}^{pkt}\frac{T_{pkt}}{2}+E_{l}\frac{\left(W_{ack}+T_{CCA}\right)T_{pkt}}{T_{l}}\;$$

(28) $$I_{2}\left(T_{c}\right)=E_{l}\frac{{\left(T_{c}-T_{pkt}\right)}^{2}}{2T_{l}}\;$$

(29) $$I_{1}\left(T_{slp}-\alpha T_{c}\right)=E_{l}\left(T_{slp}-\alpha T_{c}\right)\left(1-\frac{T_{slp}-\alpha T_{c}}{2T_{pkt}}+\frac{W_{ack}+T_{CCA}}{T_{l}}\right)$$

(30) $$I_{2}\left(T_{slp}-\alpha T_{c}\right)=\frac{E_{l}}{2T_{l}}\left(\left(T_{slp}-\alpha T_{c}\right)\left(\left(\alpha +2\right)T_{c}-T_{slp}\right)-2T_{c}T_{pkt}+T_{pkt}^{2}\right)$$

Note that $\varepsilon [E_{fd}]$ does not depend on the specific child node from which node *X* receives a packet. Now we can formulate the expectations of the energy costs in transmission and reception by recalling expressions Equations (2) and (5):

(31) $$\varepsilon \left[E_{T}\left(X\right)\right]=\left(\varepsilon \left[k\right]-1\right)E_{C}\left(X\right)+E_{C}^{\prime }\left(X\right)+E_{l}^{DAR}$$

(32) $$\varepsilon \left[E_{R,i}\left(X\right)\right]=\varepsilon \left[E_{fd}\right]+E_{rx}^{pkt}+E_{tx}^{ack}\left(d_{b,i}\left(X\right)\right)$$

In the last equation, $d_{b,i}\left(X\right)$ stands for the backward distance between node *X* and its child node $c_{i}\left(X\right)$.

Now, $\varepsilon [E_{round}\left(X\right)]$ can be simply obtained by introducing expressions Equations (31) and (32) into (15). In particular, if power control is not enabled, all energy costs in transmission become independent of distance and $\varepsilon [E_{round}\left(X\right)]$ can be formulated in a more straightforward way:

(33) $$\begin{array}{ccc} \varepsilon [E_{round}\left(X\right)] & = & \sigma \left(X\right)\varepsilon \left[E_{R}\left(X\right)\right]+\left(\sigma \left(X\right)+1\right)\varepsilon \left[E_{T}\left(X\right)\right]\\ & + & \left(\left|\frac{T_{rnd}}{T_{l}+T_{slp}}\right|-\left(\sigma \left(X\right)+1\right)\right)\left(E_{l}+E_{sleep}\right) \end{array}$$

## 5. Simulations

In this section we validate the proposed energy model by using the Avrora simulator [9]. For this purpose, we simulate a real sensor network application and compare the energy results provided by Avrora against the results provided by our model. Avrora simulates sensor applications developed in TinyOS 1.x/2.x for platforms that include an AVR microcontroller, as for instance Mica2 and MicaZ sensor nodes. However, we want to highlight the fact that the model proposed in this paper can be applied to any other hardware platform supporting a LPL implementation.

Avrora enables a complete framework of simulation of a sensor network platform running a particular TinyOS 1.x/2.x application. This simulator manages with high accuracy the time in which the actions on the physical components of the node platform take place, providing evaluators with a better understanding of their behavior. One of the strengths of Avrora is its module for the evaluation of energy consumption, which is based on the AEON energy prediction model [57]. AEON estimates the energy consumed by a node running a particular application on the basis of usage of components, times spent at each state, and their current draws. The Avrora simulator reports the overall energy consumption of each node, breaks it down into the consumptions of individual components and, in turn, into different states, by capturing all low-level events generated by the application. In our evaluations, we will focus on the energy wasted by the radio component, which accounts for the five states shown in Figure 1 (in contrast to our model which ignores transitory states).

For the simulation purpose, we have first developed a data-gathering application for TinyOS 2.x/MicaZ where every node periodically samples several sensors, composes a message from the measurements, and transmits such message towards the base station. As stated previously, MicaZ integrates a CC2420 radio [56], which is compliant with the 802.15.4 standard. Unless the developer explicitly modifies the network implementation, any application is built on top of the default network protocols, i.e., Collection Tree Protocol (CTP) and BoX-MAC-2 as routing and MAC protocols, respectively.

### 5.1. Basic Validation

We start the validation of the analytical energy model by considering the simple scenario depicted in Figure 2. Our goal is to proof that the models developed for packet transmission and packet forwarding are consistent with the results provided by Avrora, as a prior step to deal with more complex configurations.

We assume that node A executes the application that was previously described with a duty cycle DC=3%. We also set the duty cycles of node B and its parent node to 3%. The rest of parameters used in the evaluation are listed in Table 3. With these settings (DC=3% and $T_{l}$ = 5 ms), the nodes activate their radios approximately every $T_{l}+T_{slp}$ = 166.7 ms. This is the duration of a cycle with no communication activity, also called LPL interval in TinyOS context. These values are consistent with the two-state description used in the analytical model. In contrast, Table 4 shows the duration of each state in Figure 1 as measured by Avrora. As it can be noticed, there are differences between the theoretical model and the real system, but they are sufficiently small so as to accept the former with the advantage of simplification. In general, we also observed that state durations were approximately fixed, with the exception of the Power Off state. The radio remained in this state for the time until completing the nominal LPL interval, that is, $T_{LPL}$ (166.7 ms in our case). For example, upon a single radio activation with no packets to transmit or receive, the duration of the Power Off state is $T_{off}=T_{LPL}-\left(T_{down1}+T_{idle}+T_{rx}+T_{down2}\right)$, with $T_{rx}=T_{l}$. Otherwise, the remaining time depends on the number of tries that are required to successfully send/forward a packet. In this case, the term $T_{rx}$ in the previous formula would be replaced by the total duration of the duty period dedicated to receive and forward the packet, including the DELAY_AFTER_RECEIVE interval. Accordingly, the resulting value for $T_{off}$ would be lower for the same nominal $T_{LPL}$.

**Figure 7:**
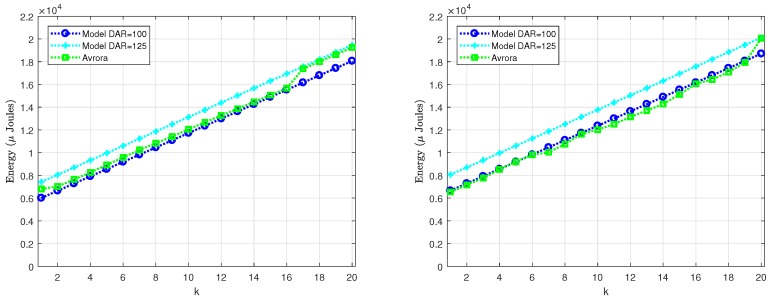
Energy consumption in μ Joules for *k* tries of a transmitter node (**left**) and a receiver and forwarding node (**right**).

Since the reporting period is typically much larger than the LPL interval ($T_{rnd}\gg T_{LPL}$), most of the times a radio activation does not entail any packet transmission or reception (see Figure 1 on the left). For the scenario under consideration, node A generates a packet only once every $T_{rnd}$, and generally it uses several tries to send this packet to node B. In turn, node B forwards the packet to its parent node, for which it also requires a certain number of tries, completely independent of those required by node A. Specifically, we performed two experiments as part of this basic validation. In the first experiment, we varied the level of asynchrony between nodes A and B, in such a way that the number of tries required by node A also varied. According to the model described in Section 4, the number of transmissions *k* ranges between 1 and α+2. For the parameter setting considered here, α=59 and hence the number of tries could vary between 1 and 61. However, in order to simplify the evaluation, we consider k∈[1,20] and, for each *k*, we compare the energy consumption provided by Avrora with the energy consumption calculated by our model. During the experiment, we realized that Avrora was not able to meet the exact duration of the DELAY_AFTER_RECEIVE (DAR) parameter, fixed in the source code to the value 100 ms and that, in general, its real duration tended to be slightly larger than 100 ms. For this reason, we evaluate the scenario using two different values of DAR, namely 100 ms and 125 ms. The results are shown in Figure 7 on the left, where the energy consumed by node A to transmit a packet is represented in terms of the number of tries (*k*). As it could be expected, energy consumption grows linearly when *k* increases. We also noticed that the deviation between the results provided by Avrora and by the model was approximately 6% in the worst case (for DAR = 125 ms), which is an acceptable value.

In the second experiment, we evaluated the energy consumed by node B to receive and forward the packet from A. In this case, we varied the level of asynchrony between node B and its parent node while keeping the time difference between nodes A and B fixed. The results are shown in Figure 7 on the right, where again a linear trend is observed. Note that the results in Figure 7 on the right are slightly larger than the figure on the left, a fact that is explained by the small extra energy required by node B to receive the packet from node A. Again, the maximum deviation between Avrora and the analytical model is below 6%.

### 5.2. Tree-Based Topologies Validation

**Figure 8:**
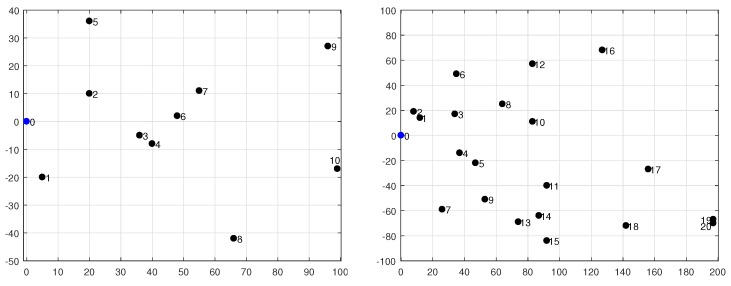
Network deployments used in the simulation tests.

In this subsection we proceed with the assessment of our analytical energy model by using realistic network topologies. Figure 8 shows the two networks under evaluation: (1) a 10-node tree-based topology deployed into a square of side L=100 m (on the left); and (2) a 20-node tree-based topology on a square of side L=200 m (on the right). Both networks use an outdoor radio range of R=75 m. We consider two values for the duty-cycle, namely DC={3,10}%. Each simulation with Avrora assumes that the application described before is run by all nodes; moreover, all of them use the same DC with the exception of the base station (node 0), as it generally lacks energy restrictions. The results provided by Avrora, namely the energy consumed by each node for a given number of communication rounds, are then compared with those obtained from the proposed energy model in order to determine the accuracy of the latter.

**Figure 9:**
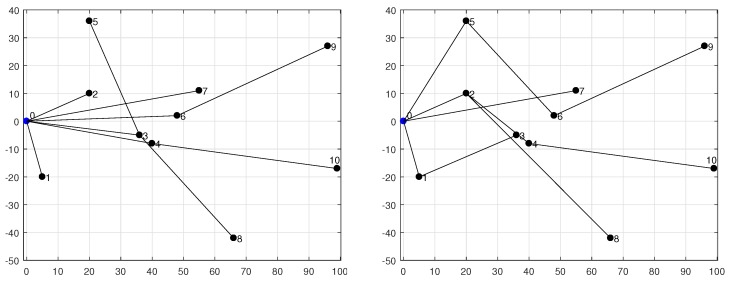
10-node topologies after simulation with DC=3% (**left**) and DC=10% (**right**).

A key parameter in our analysis is σ, which is determined for each node by network topology (recall that, in fact, σ=σ(X), with *X* representing an arbitrary node). In TinyOS-based sensor networks, the default routing protocol is CTP. CTP progressively builds a connected network by means of frequent exchanges of beacon messages between nodes, until a tree-based topology is achieved. Subsequently, the exchange of beacon messages is modulated by the temporal and spatial variation of the traffic load, as this determines the frequency of topology updates. Remind that CTP, which is responsible for collecting data from the network and sending them to a small number of sinks, was designed to achieve the objectives of agility and efficiency [33]. This means that network topology can dynamically change in response to changing traffic conditions, though this was not the case in our simulation experiments once an initial transient regime was completed. This is due to the regularity of the traffic pattern generated by the monitoring application being executed. Therefore, despite beacon messages were very frequent during an initial transient period, their impact on energy consumption became marginal as long as the simulation was evolving. As an example, Figure 9 depicts the final topologies obtained after simulating the 10-node network; one of these topologies corresponds to DC=3% (left) and the other one to DC=10% (right). In both experiments the simulation time was set large enough to allow CTP to complete the initial period of topology construction, after which we observed that the data-gathering tree remained practically constant.

**Figure 10:**
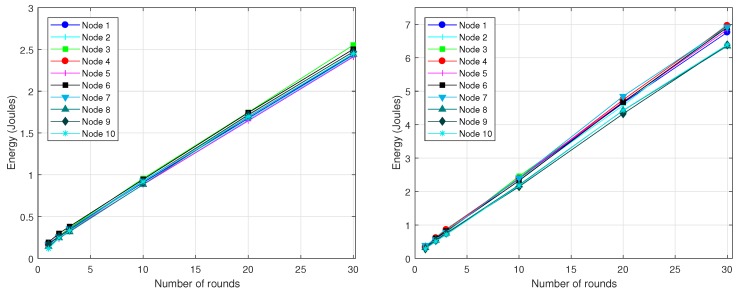
Energy consumption after the simulation of the 10-node topology, with DC=3% (**left**) and DC=10% (**right**), for 1, 2, 3, 10, 20 and 30 rounds.

At the end of each simulation experiment, Avrora reports several parameters on a per-node basis: total number of packets transmitted and received, and energy consumption. Accordingly, the results for the 10-node network are shown in Figure 10, which plots the energy consumed by every node with the exception of node 0 (base station). Only the consumption of the radio component is taken into account, both for DC=3% (on the left) and DC=10% (on the right). The *X*-axis corresponds to the number of rounds executed (1, 2, 3, 10, 20 and 30), while the *Y*-axis provides the corresponding energy consumption in Joules. As observed, the energy consumed by each node grows approximately linearly with the number of rounds, fact that is consistent with the observation that topology was almost static. We also noticed that the differences between nodes were mainly due to the combination of the parameters *k* and σ. The value of σ was very stable for each node, as the topology hardly experienced any change. Moreover, even if a topological change had taken place, this does not necessarily mean that the total number of descendants of a given node had changed as well. The other parameter that causes differences between nodes is *k*, which is unpredictable and uncontrollable as it depends on the asynchrony between the nodes in every transmitter-receiver pair. However, again with no loss of generality, we forced the stabilization of the parameter *k* of each node within the interval [1,20]; for this purpose, we used the simulator options -random-seed and -random-start, which ensure reproducible simulation results and avoid artificial cycle-level synchronization, respectively. As expected, the energy consumption grows with DC, since a larger DC means more radio activity. On the other hand, we can also observe that leaf nodes exhibit less energy consumption compared to forwarder (intermediate) nodes, fact that is due to the lesser value of σ of the former.

**Figure 11:**
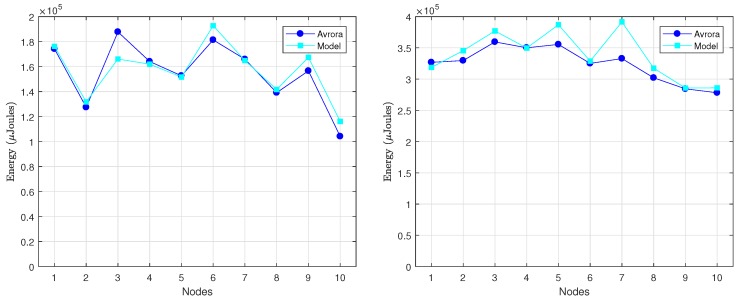
Avrora vs. energy model comparison for the 10-nodes network with DC=3% (**on the left**) and DC=10% (**on the right**).

Figure 11 shows the energy consumed per round by every node in the 10-node network, as provided by Avora and the analytical model. For the computation of the energy consumption from the analytical model, we took the specific value of *k* required by each node from the Avrora simulation (recall that *k* is an input parameter in our model). The obtained results show an average deviation of our model with regard to the Avrora simulator below 4.8% for DC=3% and 0.5% for DC=10%. The reason why the lower duty-cycle gives rise to a larger deviation is the presence of beacon messages, which are included in the energetic balance provided by Avrora but not in the analytical model: a lower duty-cycle means less communication activity and thus more impact of beacon messages in the overall evaluation of energy consumption.

**Figure 12:**
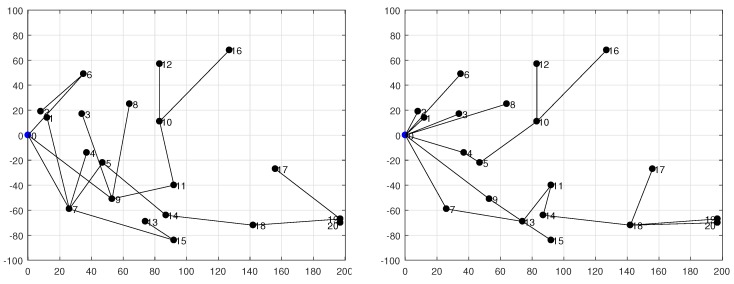
20-node topologies after simulation with DC=3% (**left**) and DC=10% (**right**).

**Figure 13:**
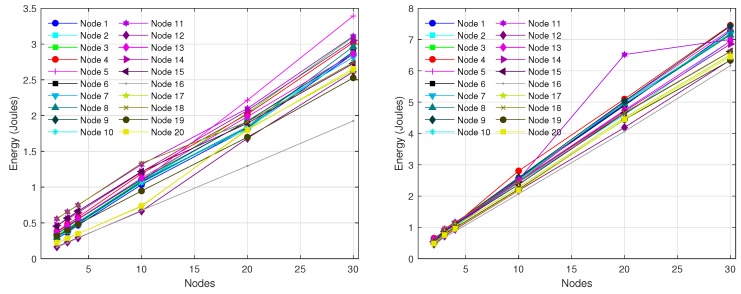
Energy consumption after the simulation of the 20-node topology, with DC=3% (**left**) and DC=10% (**right**), for 2, 3, 4, 10, 20 and 30 rounds.

The experiments done with the 20-node network produced a data-gathering tree after a longer transient period. This is because CTP requires more time to create a topology when the network size is increased. Accordingly, we set up a larger simulation time. Figure 12 shows the resulting topologies, again for DC=3% (on the left) and DC=10% (on the right). For these topologies, Figure 13 shows the energy consumption reported by Avrora for 2, 3, 4, 10, 20 and 30 communication rounds. Similarly to the results obtained for the 10-node network, we observe a linear increase of energy consumption in terms of number of rounds; however, some nodes, like node 5 in the case of DC=3% and node 11 in the case of DC=10%, exhibit a large deviation above the linear trend for some number of rounds; this is due to the fact that *k* and/or σ experienced sporadic and unpredictable changes at some time instants. While this is possible with both CTP and BoX-MAC, it does not represent the normal behavior and thus we believe that such deviations do not compromise the validity of the proposed model. As in the 10-node network, leaf nodes consume less energy than forwarding nodes.

**Figure 14:**
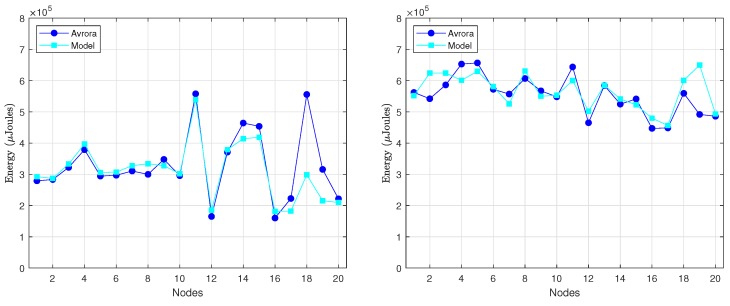
Avrora vs. energy model comparison for the 20-nodes network with DC=3% (**left**) and DC=10% (**right**).

In order to better illustrate the variation of energy consumption among nodes, Figure 14 compares, for each node, the results obtained with our model against the results of Avrora. They correspond to 2 rounds of communication and both DC=3% (on the left) and DC=10% (on the right). In this case, the average deviation obtained for the two duty-cycles was approximately the same, on the order of 3%. This is because with 20 nodes there is more communication activity in the network (σ is generally larger), fact that, in turn, diminishes the impact of beacons.

## 6. Solar Energy Harvesting Model

Another fundamental component in the analysis of an energy-harvesting system is the energy production model. Thus, for a solar-based EH-WSN, the energy that can be harvested by a solar module embedded into a sensor node needs to be characterized. Specifically, we use the energy production model proposed and validated in [58], which takes into account the relevant parameters of the solar cell, namely efficiency and surface, as well as the solar irradiance or solar intensity, defined as the amount of solar power incident per unit of surface. For the solar irradiance, the model proposed in [58] is based on data obtained from the RetScreen NASA program [59], which consists of two parameter sets for any given location in terms of its longitude and latitude coordinates: $STDHOURS_{month}$, the standard number of hours of solar light in a month, and $D_{month}$, the standard value of maximum irradiance in a month. Note that $t_{sunrise}=h-\frac{STDHOURS}{2}$ and $t_{sunset}=h+\frac{STDHOURS}{2}$ are the time instants corresponding, respectively, to the sunrise and sunset. Based on these parameters, the proposed model for the irradiance at a given time, D(t), obeys a quadratic trend:

(34) $$\begin{array}{c} D\left(t\right)=\left\{\begin{array}{cc} -\frac{{\left(t-h\right)}^{2}}{p}+D_{month}\; & \mathrm{if}\;t_{sunrise}\leq t\leq t_{sunset}\\ 0\; & \mathrm{otherwise} \end{array}\right. \end{array}$$

**Figure 15:**
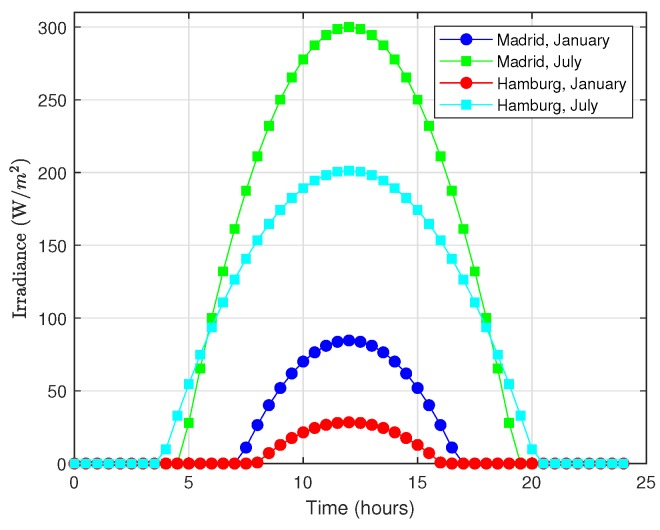
Irradiance curves for the cities of Madrid and Hamburg in January and July.

In this expression, $p=\frac{STDHOURS^{2}}{4D_{month}}$, *t* is any hour between 0:00 and 24:00 and *h* denotes the hour of maximum solar light (12:00). Though this model was further refined in [60], it predicts very accurately the solar irradiance that can be expected at given spatio-temporal coordinates. For illustration, Figure 15 shows the hourly irradiance curves for the cities of Madrid and Hamburg during the months of January and July. These curves have been obtained by using the data shown in Table 5 (extracted from the RetScreen database).

Then, the power output delivered by a solar cell of efficiency η and surface *S* can be expressed in the following way:

(35) $$P_{out}\left(t\right)=D\left(t\right)\eta S$$

Unfortunately, the efficiency of solar cells is still rather small. For instance, the efficiency of the widely used solar module KL-SUN3W is η=12.8% under standard conditions, which means that only 12.8% of the solar power absorbed is converted into electrical power.

## 7. Duty Cycle for Energy Neutral Operation

In this paper we consider the harvest-store-consume supply alternative described in [1], which consists of combining the energy harvesting subsystem (solar module) with a buffer for energy storage (rechargeable battery or supercapacitor). This latter can absorb any excess (up to a limit) of energy scavenged, which can then be available during periods of decreasing sunlight (night hours or adverse weather conditions). To start with the analysis, let us denote by $P_{c}\left(t\right)$ the power consumed by the sensor node at time *t*. Assuming that the buffer does not have any inefficiency in charging and does not leak any energy over time, the condition for energy neutral operation can be formulated as stated in [61] (the terminology has been adapted):

(36) $$E\left(t\right)=E\left(0\right)+\int_{0}^{t}P_{out}\left(u\right)\;du-\int_{0}^{t}P_{c}\left(u\right)\;du\geq 0\;\forall t\geq 0$$

In this expression, E(t) denotes the energy balance at time *t* and hence E(0) represents the initial energy stored in the buffer. Given the fact that in our case both the energy harvested and the energy consumed follow periodic patterns, the former on a daily basis (for a given month in our modelling approach), the latter on a round basis, the condition for energy neutral operation can be formulated for a one-day interval, since this is the largest period: note that typically the 24-h period is a very large multiple integer of the round duration, which is on the order of one or several minutes at most. For the same reason, we can undoubtedly assume that the energy consumed by the sensor node during a communication round is uniformly distributed over its duration, and thus the power consumption component becomes independent of time. Based on theses assumptions, we can rewrite the condition for energy neutral operation in the following way, where $T_{D}$ represents the duration of a day ($T_{D}=24$ if it is expressed in hours) and the reference to the individual node *X* has been made explicit:

(37a) $$\begin{array}{c} E\left(t,X\right)=E\left(0,X\right)+\int_{0}^{t}P_{out}\left(u\right)\;du-\int_{0}^{t}\frac{E_{round}\left(X\right)}{T_{rnd}}\;du\geq 0\;\forall t\in \left[0,T_{D}\right] \end{array}$$

(37b) $$\begin{array}{c} E\left(T_{D},X\right)=E\left(0,X\right) \end{array}$$

So, E(t,X) represents the energy available in node *X* at time *t*. Note that, for sustained operation, it would be enough that $E\left(T_{D},X\right)\geq E\left(0,X\right)$, meaning that the energy available at the node would increase from day to day. However, such a positive balance would only reflect an inefficient use of the available energy. Hence, in order to benefit the node duty cycle from any excess of harvested energy, the optimal condition is Equation (37b). This contributes to increased performance under self-sustained operation.

Let us start by evaluating the first integral in Equation (37a), which is nothing else but the energy produced by the solar cell up to time *t*, namely $E_{out}\left(t\right)=\int_{0}^{t}P_{out}\left(u\right)\;du$. Recalling Equations (34) and (35), we can easily derive the following result:

(38) $$\begin{array}{c} E_{out}\left(t\right)=\left\{\begin{array}{cc} 0 & \mathrm{if}\;t<t_{sunrise}\\ -\frac{\eta S}{3p}{\left(t-h\right)}^{3}+\eta SD_{month}t\\ +\eta S\left(\frac{{\left(t_{sunrise}-h\right)}^{3}}{3p}-D_{month}t_{sunrise}\right) & \mathrm{if}\;t_{sunrise}\leq t<t_{sunset}\\ -\frac{\eta S}{3p}{\left(t_{sunset}-h\right)}^{3}+\eta SD_{month}t_{sunset}\\ +\eta S\left(\frac{{\left(t_{sunrise}-h\right)}^{3}}{3p}-D_{month}t_{sunrise}\right) & \mathrm{if}\;t_{sunset}\leq t\leq T_{D} \end{array}\right. \end{array}$$

**Figure 16:**
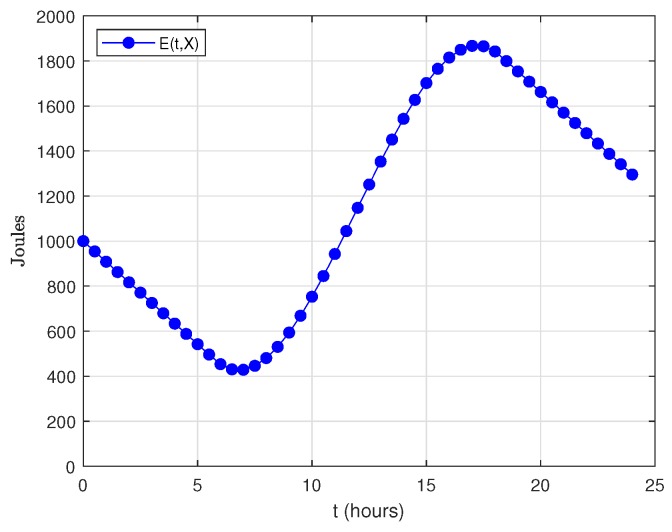
Evolution of E(t,X) during a one-day period, for DC=40%.

On the other hand, $\int_{0}^{t}\frac{E_{round}\left(X\right)}{T_{rnd}}\;du=\frac{E_{round}\left(X\right)}{T_{rnd}}t$. Figure 16 plots the evolution of E(t,X) during a one-day period. Regarding the energy consumption model, we have used the data shown in Table 3, but with $T_{rnd}=60$ s and DC=40%. The load σ(X) has been set to 30. Regarding the energy harvesting model, we have considered $D_{month}=4.87$ kWh/m${}^{2}$/day and STDHOURS=12.5 h (data corresponding to Madrid in September), E(0,X)=1000 Joules (initial energy level), a capacity of 3000 Joules and a solar cell with S=36 cm${}^{2}$ and η=11.38%. As it can be noticed, E(t,X) exhibits an oscillating behavior while condition Equation (37a) is satisfied.

Next, returning to condition Equation (37b), we can reformulate it as follows after very simple manipulations:

(39) $$E_{out}\left(T_{D}\right)=E_{round}\left(X\right)\frac{T_{D}}{T_{rnd}}$$

Note that the quotient $\frac{T_{D}}{T_{rnd}}$ is nothing else but the number of rounds per day. From Equation (39) we can derive the condition for the duty cycle of node *X*, which is a parameter contained in $E_{round}\left(X\right)$ through Equation (15). In effect, this equation can be rewritten in the following way, by simply recalling that $T_{slp}=\frac{T_{l}\left(100-DC\right)}{DC}$:

(40) $$\begin{array}{ccc} E_{round}\left(X\right) & = & \sum_{i=1}^{CH(X)}\left(\sigma \left(c_{i}\left(X\right)\right)+1\right)E_{R,i}\left(X\right)+\left(\sigma \left(X\right)+1\right)E_{T}\left(X\right)\\ & + & \left(\left|\frac{T_{rnd}}{T_{l}+\frac{T_{l}\left(100-DC\right)}{DC}}\right|-\left(\sigma \left(X\right)+1\right)\right)\left(E_{l}+E_{sleep}\right) \end{array}$$

**Figure 17:**
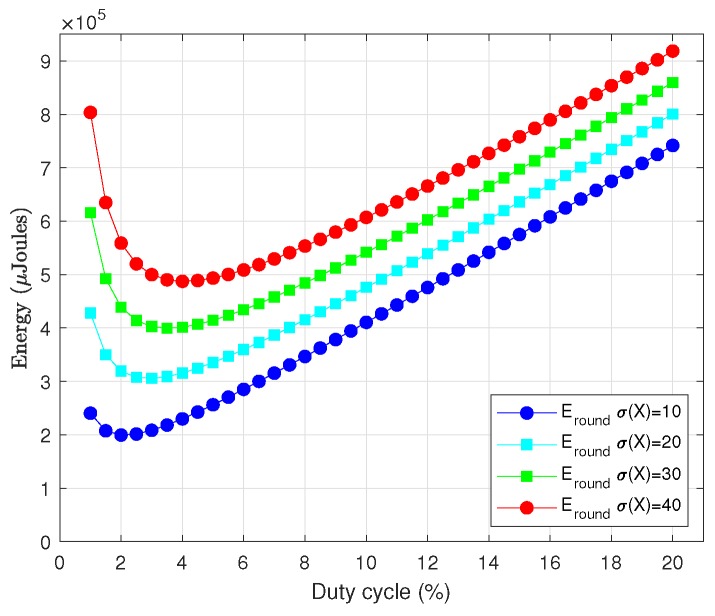
Energy consumption per round in terms of the duty cycle.

**Figure 18:**
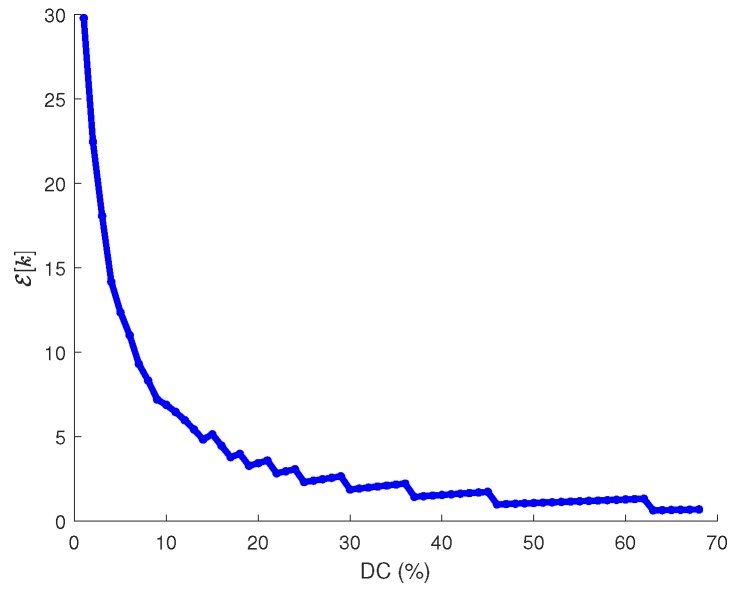
Expected number of tries in terms of the duty cycle.

In order to simplify the process of obtaining a closed-form expression for DC, it is convenient first to analyze the dependence of $E_{round}\left(X\right)$ on this parameter. Figure 17 shows $E_{round}\left(X\right)$ in terms of DC for different values of σ(X), $T_{rnd}=60$ s and the rest of parameters as given in Table 3. As it can be noticed, all curves exhibit a linear trend from relatively small values of DC. Additionally, recall from Section 1 that, whereas in battery-operated sensor networks the primary goal was to keep energy consumption as low as possible, in the case of EH-WSN the focus is on enhancing performance as long as energy neutral operation is preserved. Enhancing performance means enlarging the duty cycle of nodes as much as possible, as demonstrated in Figure 18, which shows the decrease of ε[k] as DC increases (note that smaller values of ε[k] imply higher throughput and lower delays). On the other hand, large values for DC (on the order of 40–50%) clearly correspond to the linear region of all curves in Figure 17. Thus, we can definitely make use of the linear approximation of $E_{round}\left(X\right)$ in terms of DC for sufficiently large values of the latter. After performing several manipulations, the resulting expression is as follows:

(41) $$E_{round}\left(X\right)≅I_{rx}VT_{rnd}\frac{DC}{100}+\left(\sigma \left(X\right)+1\right)E_{l}^{DAR}$$

**Figure 19:**
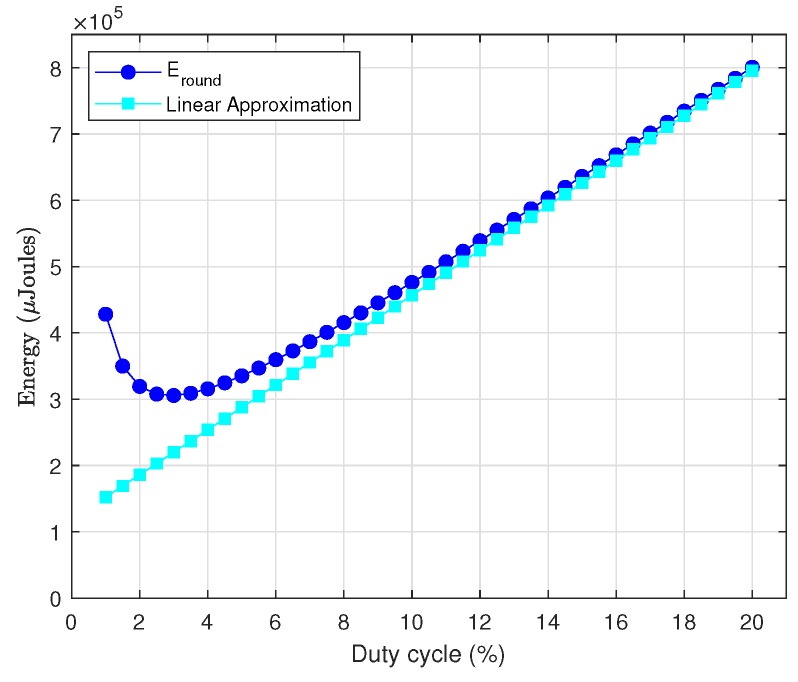
Linear approximation to $E_{round}\left(X\right)$ from moderately low values of DC, for σ(X)=20.

We have validated this approximation for several parameter combination. Figure 19 shows the results of one of the tests, which corresponds to most of data contained in Table 3 and σ(X)=20. In all cases, we could observe the same behavior: the straight line approaches very accurately the real function from moderately low values of DC onwards.

We can now introduce the linear approximation into Equation (39) and isolate DC to obtain the following result:

(42) $$\frac{DC}{100}=\frac{E_{out}\left(T_{D}\right)}{I_{rx}VT_{D}}-\frac{\left(\sigma \left(X\right)+1\right)E_{l}^{DAR}}{I_{rx}VT_{rnd}}$$

**Figure 20:**
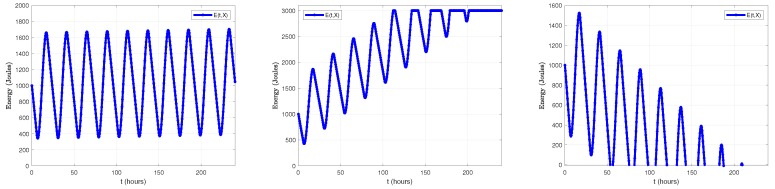
Evolution of E(t,X) for DC=46% (**left**), for DC=40% (**center**) and for DC=50% (**right**).

Note that, in fact, this equation defines a threshold value for DC: if the real DC is larger, then the energy available at the node, E(t,X), exhibits a decreasing trend until the node eventually reaches a blocking status; in contrast, if it is smaller, the trend is increasing but evolving towards a flat as the node energy approaches the capacity of the energy buffer. Though this latter situation is feasible from an operational point of view, it reflects a downward dimensioning of the duty cycle that entails some unnecessary performance degradation. The three situations are described in Figure 20, for which we have reused the data of Figure 16 (except the duty cycle). Specifically, for such dataset the duty cycle obtained by applying Equation (42) is DC≅46%. Accordingly, Figure 20 shows the evolution of E(t,X) corresponding to DC=46%, DC=40% and DC=50%. Note that the evolution of E(t,X) has been extended to 10 days in the three curves, in order to clearly show their trends and the effects of the energy capacity in the case of Figure 20 located on the center.

The last part of the analysis consists of determining a minimum value for the initial energy level, namely E(0,X), such that condition Equation (37a) is satisfied for the one-day period. Note that, in our modelling approach, if this condition holds and the duty cycle is less than or equal to the value obtained from Equation (42), then sustained operation is guaranteed. Note also that condition Equation (37a) is fulfilled as long as the minimum of E(t,X) along the one-day period is greater than or equal to zero. Thus, the next step is to determine such minimum by setting $\frac{dE(t,X)}{t}=0$. In fact, this process leads to the calculation of both the maximum and minimum of E(t,X) with the help of the second derivative. Let $T_{min}$ and $T_{max}$ denote, respectively, the time instants within a one-day period for which the minimum and maximum of E(t,X) take place—recall Figure 16. The results are as follows:

(43) $$T_{min}=h-\frac{STDHOURS}{2}\sqrt{1-\frac{E_{round}\left(X\right)}{\eta ST_{rnd}D_{month}}}$$

(44) $$T_{max}=h+\frac{STDHOURS}{2}\sqrt{1-\frac{E_{round}\left(X\right)}{\eta ST_{rnd}D_{month}}}$$

For the calculation of $E_{round}\left(X\right)$, either Equation (40) or its linear approximation Equation (41) can be used. Next, by imposing $E(T_{min},X)\geq 0$ and performing some manipulations, we can end up with the following result for the initial energy:

(45) $$E\left(0,X\right)\geq \frac{E_{round}\left(X\right)}{T_{rnd}}T_{min}-E_{out}\left(T_{min}\right)$$

Equations (42) and (45) represent the final results of this paper for the modelling approach and assumptions that have been adopted. Certainly, real weather conditions often deviate from the periodic behavior exhibited by the prediction model proposed in Section 6, and in addition other unexpected changes may also take place (for instance, topological updates). However, we believe that those equations can be used as starting points to develop adaptive schemes that also rely on the capabilities of nodes of measuring their own energy level. We emphasize this task as an issue for further research in the next section.

## 8. Conclusions and Future Work

In this paper, we have obtained closed-form expressions for the duty cycle and initial energy storage that guarantee self-sustained operation in TinyOS solar-based EH-WSN devoted to periodic monitoring. To achieve these results, first we have developed an energy consumption model, whose deviation from simulation estimates is 4.8% in the worst case. Then, we have formulated the condition for energy neutral operation by combining an approximate version of the energy consumption model with a well-known solar irradiance model. This model assumes a periodic pattern for the irradiance level that is based on the average prediction at given spatial and temporal coordinates. The approximate energy consumption model omits the details of the LPL mechanism implemented in TinyOS sensor nodes, while at the same time it retains the essential components. For this reason, we postulate that the obtained formulation can be extended to other software (with corresponding hardware) platforms, and thus it can be used as a starting point to develop adaptive schemes that dynamically adjust the duty cycle of nodes according to changing traffic and weather conditions. We leave these issues for further research. We also suggest that the energy-harvesting model considered in this paper be refined according to the geographic latitude and meteorological conditions of the deployment scenario. 

## Figures and Tables

**Table 1:** Characterization of the number of tries (*k*) in the process of sending a packet.

Time Intervals (for the Backend)	*k*	Probability
[0,$T_{CCA}$) U [$T_{CCA}+T_{slp}$, $T_{l}+T_{slp}$]	1	$\frac{T_{l}}{T_{l}+T_{slp}}$
[$T_{CCA}$, $T_{CCA}+T_{c}$)	2	$\frac{T_{c}}{T_{l}+T_{slp}}$
[$T_{CCA}+T_{c}$, $T_{CCA}+2T_{c}$)	3	$\frac{T_{c}}{T_{l}+T_{slp}}$
[$T_{CCA}+2T_{c}$, $T_{CCA}+3T_{c}$)	4	$\frac{T_{c}}{T_{l}+T_{slp}}$
[$T_{CCA}+3T_{c}$, $T_{CCA}+4T_{c}$)	5	$\frac{T_{c}}{T_{l}+T_{slp}}$
…	…	…
[$T_{CCA}+\left(\alpha -1\right)T_{c}$, $T_{CCA}+\alpha T_{c}$)	α+1	$\frac{T_{c}}{T_{l}+T_{slp}}$
[$T_{CCA}+\alpha T_{c}$, $T_{CCA}+T_{slp}$)	α+2	$\frac{T_{slp}-\alpha T_{c}}{T_{l}+T_{slp}}$

**Table 2:** Characterization of $(E_{fd}$) in terms of the transmitter-receiver asynchrony.

Time Intervals (var. t)	Subintervals (var. t’)		$E_{fd}$
$\left[0,T_{CCA}\right)$	$E_{l}\frac{T_{CCA}-t}{T_{l}}$		
$\left[T_{CCA},T_{CCA}+T_{c}\right)$	$\left[0^{+},T_{pkt}\right)$		$E_{rx}^{pkt}\frac{T_{pkt}-t^{\prime }}{T_{pkt}}+E_{l}\frac{W_{ack}+T_{CCA}}{T_{l}}$
	$\left[T_{pkt},T_{c}^{-}\right)$		$E_{l}\frac{T_{c}-t^{\prime }}{T_{l}}$
$\left[T_{CCA}+T_{c},T_{CCA}+2T_{c}\right)$	$\left[0^{+},T_{pkt}\right)$		$E_{rx}^{pkt}\frac{T_{pkt}-t^{\prime }}{T_{pkt}}+E_{l}\frac{W_{ack}+T_{CCA}}{T_{l}}$
	$\left[T_{pkt},T_{c}^{-}\right)$		$E_{l}\frac{T_{c}-t^{\prime }}{T_{l}}$
$\left[T_{CCA}+2T_{c},T_{CCA}+3T_{c}\right)$	$\left[0^{+},T_{pkt}\right)$		$E_{rx}^{pkt}\frac{T_{pkt}-t^{\prime }}{T_{pkt}}+E_{l}\frac{W_{ack}+T_{CCA}}{T_{l}}$
	$\left[T_{pkt},T_{c}^{-}\right)$		$E_{l}\frac{T_{c}-t^{\prime }}{T_{l}}$
…	…		…
$\left[T_{CCA}+\left(\alpha -1\right)T_{c},T_{CCA}+\alpha T_{c}\right)$	$\left[0^{+},T_{pkt}\right)$		$E_{rx}^{pkt}\frac{T_{pkt}-t^{\prime }}{T_{pkt}}+E_{l}\frac{W_{ack}+T_{CCA}}{T_{l}}$
	$\left[T_{pkt},T_{c}^{-}\right)$		$E_{l}\frac{T_{c}-t^{\prime }}{T_{l}}$
$\left[T_{CCA}+\alpha T_{c},T_{CCA}+T_{slp}\right)$	if ($T_{slp}\leq \alpha T_{c}+T_{pkt}$)	$\left[0^{+},T_{slp}-\alpha T_{c}\right)$	$E_{rx}^{pkt}\frac{T_{pkt}-t^{\prime }}{T_{pkt}}+E_{l}\frac{W_{ack}+T_{CCA}}{T_{l}}$
	else	$\left[0^{+},T_{pkt}\right)$	$E_{rx}^{pkt}\frac{T_{pkt}-t^{\prime }}{T_{pkt}}+E_{l}\frac{W_{ack}+T_{CCA}}{T_{l}}$
		$\left[T_{pkt},T_{slp}-\alpha T_{c}\right)$	$E_{l}\frac{T_{c}-t^{\prime }}{T_{l}}$
$\left[T_{CCA}+T_{slp},T_{l}+T_{slp}\right]$	$E_{l}\frac{T_{l}+T_{slp}+T_{CCA}-t}{T_{l}}$		

**Table 3:** Parameters used in the validation of the analytical model.

Symbol	Description	Value
BW	802.15.4 Bandwidth	250 Kbps
$L_{pkt}$	Length of data packets	41 Bytes
$T_{pkt}$	Duration of data packets	$\frac{L_{pkt}}{BW}$
$L_{ack}$	Length of acknowledgement packets	17 Bytes
$T_{ack}$	Duration of acknowledgement packets	$\frac{L_{ack}}{BW}$
$T_{CCA}$	CCA interval	0.4 ms
$W_{ack}$	Waiting time for ACK	1 ms
$T_{c}$	Transmission cycle	2.712 ms
$T_{rnd}$	Round time (reporting period)	30 s
DC	Duty cycle	3%, 10%
$T_{l}$	DUTY_ON_TIME	5 ms
DAR	DELAY_AFTER_RECEIVE	100 ms
*B*	Battery	2500 mAh
*V*	Voltage	3 V
$I_{off}$	Current draw in Power Off	$0.2\cdot 10^{-6}$ A
$I_{down}$	Current draw in Power Down	$20\cdot 10^{-6}$ A
$I_{idle}$	Current draw in Idle	$0.426\cdot 10^{-3}$ A
$I_{tx}$	Current draw in TX (at 0 dbM)	0.0174 A
$I_{rx}$	Current draw in RX	0.0188 A

**Table 4:** Duration of each CC2420 radio state on a radio activation with no incoming packets.

State	Notation	Duration (ms)
Power Down 1	$T_{down1}$	1.680750
Idle	$T_{idle}$	0.146625
RX	$T_{rx}$	4.742875
Power Down 2	$T_{down2}$	0.000250
Power Off	$T_{off}$	To be adjusted

**Table 5:** Daylight hours and solar irradiance parameters for Madrid (latitude: 40.437944; longitude: −3.679536) and Hamburg (latitude: 53.558869; longitude: 9.927821) during the months of January and July.

City/Month	*STDHOURS* (Hours)	$D_{\mathrm{month}}$ (kWh/m${}^{2}$/day)
Madrid/January	9.65	2.03
Madrid/July	14.70	7.20
Hamburg/January	8.10	0.68
Hamburg/July	16.40	4.83
